# Global Research Status and Trends of Femoral Neck Fracture Over the Past 27 Years: A Historical Review and Bibliometric Analysis

**DOI:** 10.3389/fsurg.2022.875040

**Published:** 2022-06-14

**Authors:** Peng Peng, Fangjun Xiao, Xiaoming He, Weihua Fang, Jiewen Huang, Bin Wang, Yiwen Luo, Qinwen Zhang, Ying Zhang, Wei He, Qiushi Wei, Mincong He

**Affiliations:** ^1^ The First Clinical Medical School, Guangzhou University of Chinese Medicine, Guangzhou, China; ^2^ The Third Clinical Medical School, Guangzhou University of Chinese Medicine, Guangzhou, China; ^3^ Guangdong Research Institute for Orthopedics & Traumatology of Chinese Medicine, Guangzhou, China; ^4^ Department of Orthopaedics, The Third Affiliated Hospital, Guangzhou University of Chinese Medicine, Guangzhou, China; ^5^ Medical Center of Hip, Luoyang Orthopedic-Traumatological Hospital (Orthopedics Hospital of Henan), China

**Keywords:** femoral neck fracture, bibliometric analysis, visualization, research trends, hotspots

## Abstract

**Background:**

Femoral neck fracture (FNF) is a commonly encountered injury in orthopedic practice, and many studies have been conducted in this field. However, no bibliometric studies regarding the global research trend concerning FNF have been performed. This study aims to analyze the knowledge framework, research hotspots, and theme trends in the field of FNF research.

**Methods:**

The scientific outputs related to FNF from 1994 to 2021 were retrieved from the Web of Science Core Collection. Three bibliometric tools were used for this study. The main analyses include publication and citation counts, contributions of countries, institutions, authors, funding agencies and journals, and clustering of keywords.

**Results:**

In total, 3,553 articles were identified. The annual publication counts of FNF showed an ascending tendency as a whole. The United States has the most prominent contributions, with the most number of publications and the highest H-index. Karolinska Institutet devoted the most in this domain. Professors Bhandari M, Schemitsch EH, Frihagen F, Parker MJ, and Rogmark C were the core authors in this field. The most productive journal was *Injury International Journal of the Care of the Injured.* Keywords were divided into four clusters: epidemiology and mortality, fracture prevention, internal-fixation and risk factors, and hip replacement. A trend of balanced and diversified development existed in these clusters. Keywords with the ongoing bursts, including “outcome,” “reoperation,” “complication,” “revision,” “displaced intracapsular,” “fracture,” and “adult,” are considered as the research hotspots in the future and deserve more attention.

**Conclusions:**

The management of FNF in young patients is drawing more attention from orthopedic surgeons, and it is expected that these research topics may continue to be the research hotspots and focus in the near future.

## Introduction

Femoral neck fracture (FNF) is a commonly encountered injury in orthopedic practice with a high rate of morbidity and mortality (1). Patients with FNF have been on the rise in the last three decades, and it is expected the incidence rates of FNF will be continuously increasing in the coming 30 years (2, 3). FNF often occurs in elderly patients as a result of low-energy falls (4, 5). FNF is rare in young people but of high clinical relevance due to the complexity of complications and surgical challenges (6, 7). In addition to the significant effect on health, FNF represents a sizable burden to society due to high healthcare-related costs (8, 9).

Currently, the aim of treatment of FNF is to achieve early patient mobilization, reduce the risk of complications, and improve patient’s outcomes. The surgical methods are various according to the stability and orientation of the fracture and patient’s factors, mainly including arthroplasty and internal-fixation (10–12). In contrast, conservative treatment may be a better option for those with poor general conditions accompany with excessive surgical risk (13). In the past few decades, the number of studies has been growing outputs. The new information concerning internal-fixation and survival and prognosis analysis continues to appear. Thus, it is necessary to analyze the development trends and research hotspots of FNF.

Bibliometric analysis is a powerful tool for quantitative analysis of articles or review within a specific field through employing mathematical, statistical, and other econometric methods (14). It has been widely used in medical fields including obstetrics and gynecology, orthopedics, nephrology, neurosurgical, and rheumatology (15–19). Therefore, this study aimed to use a bibliometric method to analyze the knowledge framework, research hotspots, and theme trends in the field of FNF research for the first time.

## Materials and Methods

### Data Acquisition and Retrieval Strategies

**Figure 1:**
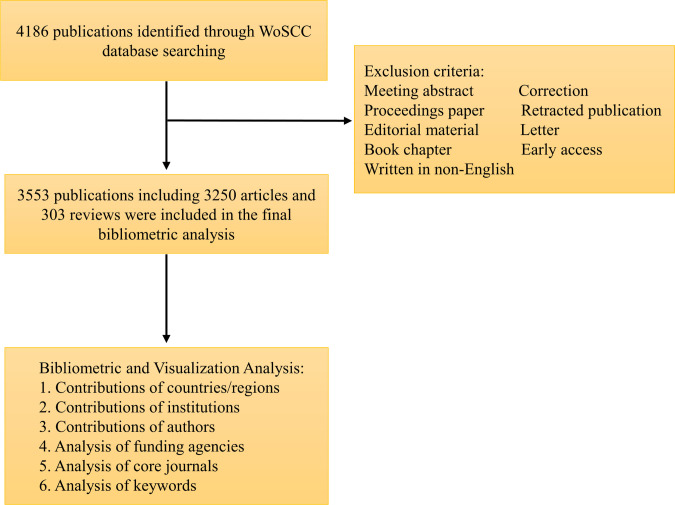
Flowchart for the selection of literature included in this study.

We conducted a literature search on the Web of Science Core Collection (WoSCC). The database includes more than 12,000 international academic journals of the greatest impact and quality and is one of the most frequently used databases in previous bibliometrics studies (20, 21). The retrieval strategy was as follows: TS = (“femoral neck fracture” OR “femur neck fractures” OR “femur neck fracture” OR “femoral neck fractures”). We included publications from 1994 to 2021 (December 31, 2021), and the language was restricted to English. The document types were limited to original articles and reviews. Figure 1 presents the literature search and selection processes. All the above operations were performed within 1 day (January 4, 2022).

### Data Extraction and Collection

All retrieved literature was downloaded and exported in text format. Information on the selected articles including the number of publications and citations, titles, authors, affiliations, countries, keywords, journal, publication year, average citation per item (ACI), and H-index. Microsoft Office Excel 2019 (Microsoft Corporation, Redmond, Washington, USA) was used to collect and rank all the publication characteristics. Manual screening and processing of synonymous keywords were performed by two independent investigators. GraphPad Prism 8.0 (GraphPad Software Inc.) was also applied to analyze data and create graphs.

### Bibliometric and Visualized Analysis

CiteSpace V (version 5.7. R5) (22), VOSviewer (version 1.6.16) (23) and an online analytical platform (https://bibliometric.com/) were used to perform this bibliometric analysis and data visualization. In this study, we used the default parameters in CiteSpace and VOSviewer. VOSviewer was applied to conduct the bibliometric analysis and visualization research co-citation of journals, co-authorship of countries, and keyword co-occurrence. In the network map created by VOSviewer, various nodes are labeled with different elements including countries, journals, and keywords. The size of the nodes reflected the number of publications, citations, or occurrences. The links between nodes represented the associations including co-authorship or co-citation. The weighted total link strength (TLS) was used to measure the strength of the links between the selected nodes (23).

CiteSpace was utilized to conduct cooperation and co-citation analyses of institutions or authors, the dual-map overlay of scientific journals, and burst keywords. In the network maps, the nodes represent the items being analyzed. Betweenness centrality (BC) is a crucial parameter that could measure the scientific importance of the nodes in the network, and nodes with high betweenness centrality (BC ≥ 0.1) are usually indicated by purple rings, and also connect more links (24). In terms of the clusters view map, cited authors of similar categories were gathered in a cluster. The bursts of keywords are often used to detect new research trends in the field. Through detailed analysis using CiteSpace, we have selected the top 30 keywords with the strongest citation bursts.

## Results

### Global Publication and Citation Trend

**Figure 2:**
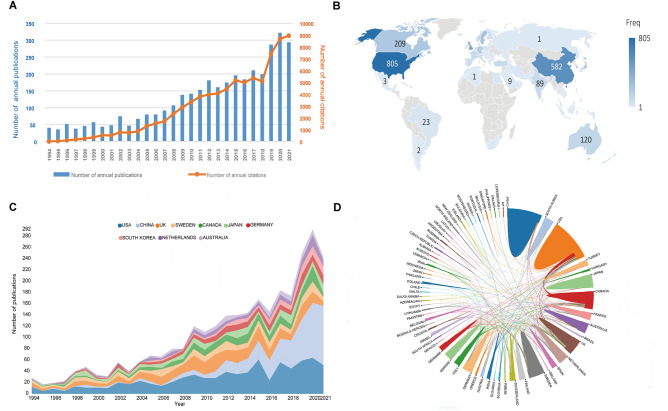
(**A**) The number of annual publications and citations on FNF research from 1994 to 2021. (**B**) A world map displaying the contribution of countries in FNF research. Countries were coded with different colors depending on the number of publications. (**C**) The annual number of publications in the top 10 most productive countries from 1994 to 2021. The width of the line in different colors reflects the changing trend of annual publications in different countries at different time points. (**D**) The cooperation map of countries/regions involved in FNF research. The thickness of each line reflects the tightness of cooperation, and a thicker line indicates a stronger cooperation.

In total, 3553 publications (3250 articles and 303 reviews) were included in this study (Figure 1). Trends in the number of annual publications and citations are presented in Figure 2A. As can be seen, the annual number of publications related to FNF showed an ascending tendency as a whole. The number of publications has increased from 41 (1994) to 294 (2021), and almost 37.0% of them (1,313 papers) were published over the last 5 years. When it comes to the number of citations, the cumulative total citations for all publications were 79,711 times (59,626 times after the removal of self-citations), with an average of 22.44 times per publication. Similar to the change in publications, there is also an ascending trend in the number of citations yearly.

### Contributions of Countries/Regions

**Figure 3:**
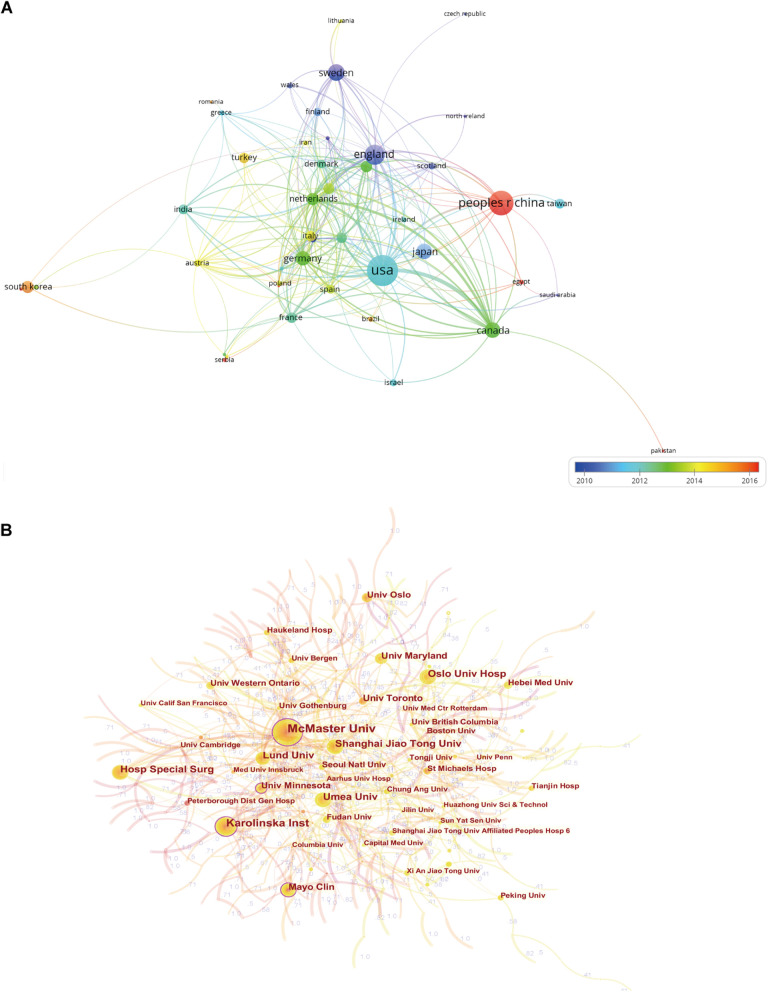
(**A**) The co-authorship map of countries/regions involved in FNF research (generated by VOSviewer). Each node represents a different country, and the node size is proportionate to the number of publications. The color of each node represents the average appearing year (AAY) of the country, depending on the color gradient at the bottom right. (**B**) The cooperation network map of institutions involved in FNF research (generated by CiteSpace). The nodes with a high betweenness centrality (BC)-value (≥0.1) are indicated by purple rings.

A total of 82 countries/regions contributed to this research field (Figure 2B), and the top 10 most productive countries are shown in Table 1. The USA had the largest number of publications and the highest value of H-index. Publications in the Netherlands had the highest average number of citations (48.9). The variation trend in the annual publication numbers from the top 10 productive countries from 1994 to 2021 is illustrated in Figure 2C. The visualization map of research collaboration between countries/regions is presented in Figure 2D. In this network, the USA collaborated most closely with China, Canada, Japan, and the UK. A country co-authorship overlay visualization map is generated by VOSviewer (Figure 3A). Of the 45 countries/regions with a minimum number of five publications, used to construct the co-authorship network, the top three with the largest TLS were listed as follows: USA, Canada, and UK.

**Table 1:** The top 10 countries and institutions with the most publications related to FNF research.

Rank	Countries	Counts	H-index	ACI	Institutions	Countries	Counts	H-index	ACI
1	USA	805	76	31.58	Karolinska Institutet	Sweden	89	32	32.73
2	China	582	29	7.4	McMaster University	Canada	82	27	34.1
3	UK	431	57	32.14	University of California System	USA	72	21	34.76
4	Sweden	240	51	32.88	University of Oslo	Norway	71	24	22.38
5	Canada	209	42	30.86	Lund University	Sweden	70	27	30.27
6	Japan	200	33	19.89	University of Toronto	Canada	65	25	43.69
7	Germany	173	33	21.38	Skane University Hospital	Sweden	60	25	30.43
8	Netherlands	128	32	48.9	Shanghai Jiao Tong University	China	57	12	6.84
9	South Korea	125	18	9.35	Umea University	Sweden	55	23	42.56
10	Australia	120	33	36.01	Hospital for Special Surgery	USA	45	15	25.13

*ACI, average citation per item. Publications from Taiwan and Hong Kong were assigned to China, and those from England, Northern Ireland, Scotland, and Wales were reclassified to the UK.*

### Contributions of Institutions

In terms of research institutions, only the top 10 are specifically listed in Table 1. Among them, Karolinska Institutet held the largest number of publications. The H-index in Karolinska Institutet 32 exceeded other institutions, ranking first. While in terms of ACI, the University of Toronto had the most average number of citations (43.69). A cooperation visualization map of the FNF research network is generated by CiteSpace and presented in Figure 3B. The inter-institutional collaboration was relatively low and mainly conducted in Canadian and American institutions. McMaster University, Karolinska Institutet, Mayo Clinic, and University of Minnesota were the only four institutions with the BC values greater than 0.1.

### Contributions of Authors

**Figure 4:**
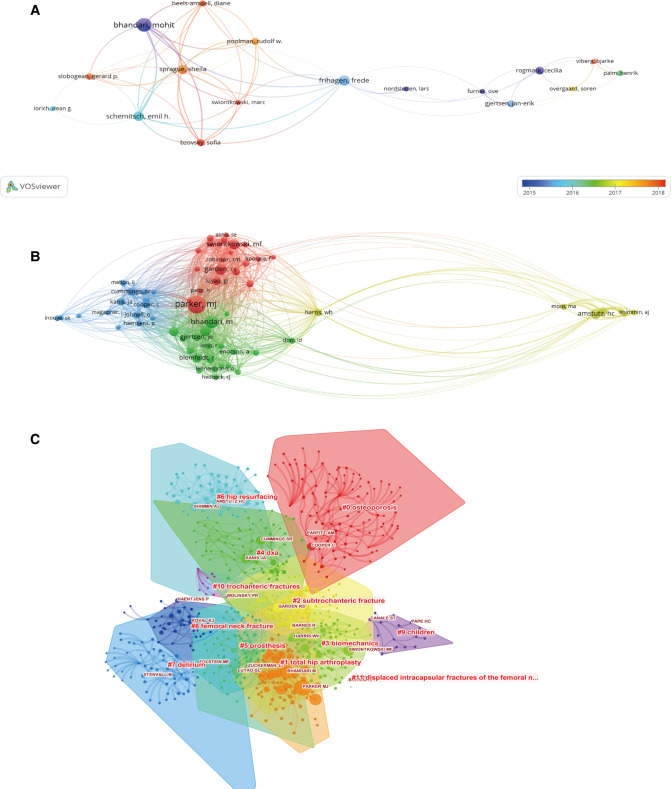
(**A**) Author co-authorship overlay visualization map generated by VOSviewer. Explanations are the same as in Figure 3(A). (**B**) Network visualization map of author co-citation analysis generated by the VOSviewer. Each node represents a different author, and the node size is proportional to the number of citations. (**C**) In the cluster map, cited authors with similar categories were gathered in a cluster. Twelve clusters with different research topics were formed, reflected in different colors on the map (generated by CiteSpace).

The top 10 authors who contributed and cooperated most are presented in Table 2 separately. Bhandari M from McMaster University was the author with the most publications of 78, followed by Schemitsch EH and Frihagen F. Parker MJ with 1281 co-citations, ranked first among the top 10 co-cited authors, followed by Bhandari M, Rogmark C, and Garden RS. Figure 4A is an overlay visualization map for author co-authorship analysis with minimum publications of 10. In the network map, Bhandari M, Schemitsch EH, and Frihagen F were located at the central position of the cooperating clusters with the largest TLS. Based on co-citation analysis performed with VOSviewer (Figure 4B), we defined “core author” as one who had acquired at least 100 citations. The top three authors with the largest TLS were Parker MJ, Bhandari M, and Rogmark C. Meanwhile, the co-citation relationships between authors were analyzed by CiteSpace via creating network visualization maps. As for the cluster view of the co-citation map (Figure 4C), the silhouette value of clusters #0 to #11 was from 0.818 to 0.988, suggesting good homogeneity. Research categories of authors were divided into 12 clusters.

**Table 2:** The top 10 most productive and co-cited authors in FNF research.

RANK	Author	Counts	H-index	ACI	Co-cited author	Citation counts	TLS
1	Bhandari M	78	28	36.55	Parker MJ	1,281	1194.31
2	Schemitsch EH	47	18	34.66	Bhandari M	685	644.04
3	Frihagen F	45	20	27.76	Rogmark C	487	474.74
4	Sprague S	39	17	29.26	Garden RS	485	473.15
5	Rogmark C	30	19	30.73	Amstutz HC	443	388.56
6	Tidermark J	27	23	67	Swiontkowski MF	440	420.28
7	Gustafson Y	25	21	69.52	Blomfeldt R	353	345.28
8	Parker MJ	25	18	62.16	Tidermark J	347	325.71
9	Poolman RW	25	11	19.68	Gjertsen JE	310	299.56
10	Bzovsky S	23	6	7.96	Cummings SR	293	280.60

*ACI, average citation per item; TLS, total link strength.*

### Analysis of Funding Agencies

**Figure 5:**
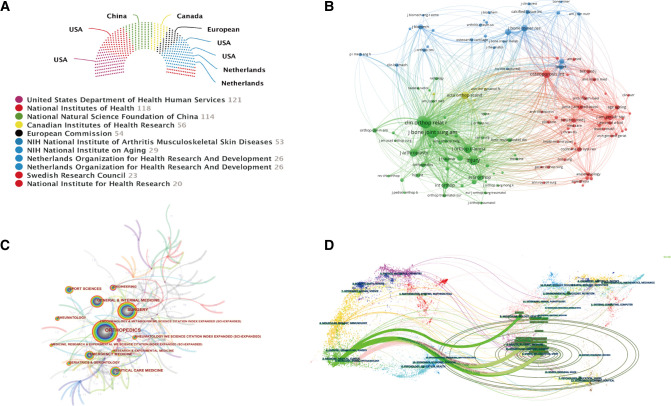
(**A**) Top 10 related funding agencies for the support of FNF research. (**B**) Journal co-citation analysis by using VOSviewer. Each node represents a different journal, and the node size is proportional to the number of citations. (**C**) Co-occurring network map of subject categories on FNF research by using CiteSpace. (**D**) The dual-map overlay of the journals on FNF research by using CiteSpace.

The United States Department of Health and Human Services funded the most publications (121; 3.4%), followed by the National Institutes of Health (118; 3.6%) and the National Natural Science Foundation of China (114; 3.2%) (Figure 5A). Five funding institutions in the United States provided funding for publications of the FNF research. The remaining funding institutions were located in China, Canada, the Netherlands, European Union, and Sweden.

### Analysis of Core Journals and Subject Categories

Table 3 shows the information on the top 10 journals. *Injury International Journal of the Care of the Injured* (291, 8.2%) had the highest number of outputs. *Journal of Bone and Joint Surgery-American Volume* had the largest impact factor of 4,578. According to the JCR 2020 standards, the top 10 most prolific journals were classified as Q1 in 2, Q2 in 4, Q3 in 2, and Q4 in 2. VOSviewer software was used to analyze the co-citation of journals. As shown in Figure 5B, 123 journals with a minimum of 100 citations were included. The top three journals with the largest TLS were listed as follows: *Journal of Bone* and *Joint Surgery-American Volume*, *Clinical Orthopaedics and Related Research*, and *Journal of Bone and Joint Surgery-British Volume*. The top 10 subject categories ranked by the number of publications are illustrated in Figure 5C. Orthopedics, Surgery, and Emergency Medicine were the top three subject categories that received the most attention in this field. In addition, we conducted a dual-map overlay of the journals on FNF research by using CiteSpace. As can be seen from Figure 5D, there were five core citation paths in the dual-map including one orange path, three green paths, and two pink paths.

**Table 3:** The top 10 journals with the most publications in FNF research.

RANK	Journal title	Counts (N)	Percentage (N/3,553, %)	IF (2020)	JCR (2020)	H-index	ACI
1	*Injury International Journal of the Care of the Injured*	291	8.2	2.586	Q3	41	18.86
2	*Journal of Orthopaedic Trauma*	188	5.3	2.512	Q3	37	20.81
3	*Clinical Orthopaedics and Related Research*	146	4.1	4.176	Q1	47	39.38
4	*Journal of Arthroplasty*	139	3.9	4.757	Q2	29	19.52
5	*Archives Of Orthopaedic and Trauma Surgery*	116	3.3	3.067	Q2	23	14
6	*International Orthopaedics*	109	3.1	3.075	Q2	25	19.04
7	*Journal of Bone and Joint Surgery-American Volume*	94	2.7	5.284	Q1	39	58.97
8	*BMC Musculoskeletal Disorders*	93	2.6	2.362	Q4	19	10.7
9	*Acta Orthopaedica*	81	2.3	3.717	Q2	27	27.25
10	*Hip International*	77	2.2	2.135	Q4	11	5.44

*ACI, average citation per item.*

### Keyword Analysis of Research Hotspots

**Figure 6:**
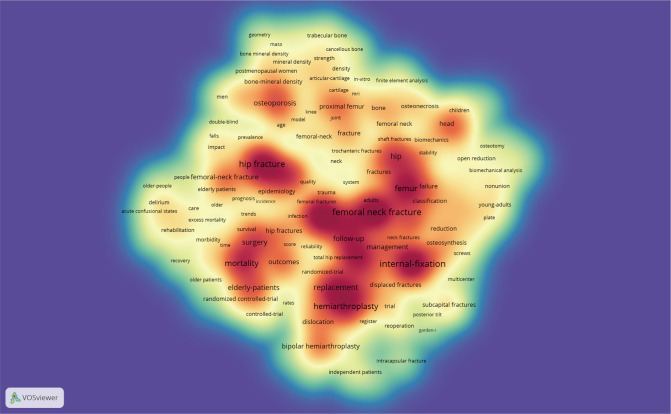
The density visualization map of keyword co-occurrence analysis (generated by VOSviewer). The darker the color, the higher the keyword density.

**Figure 7:**
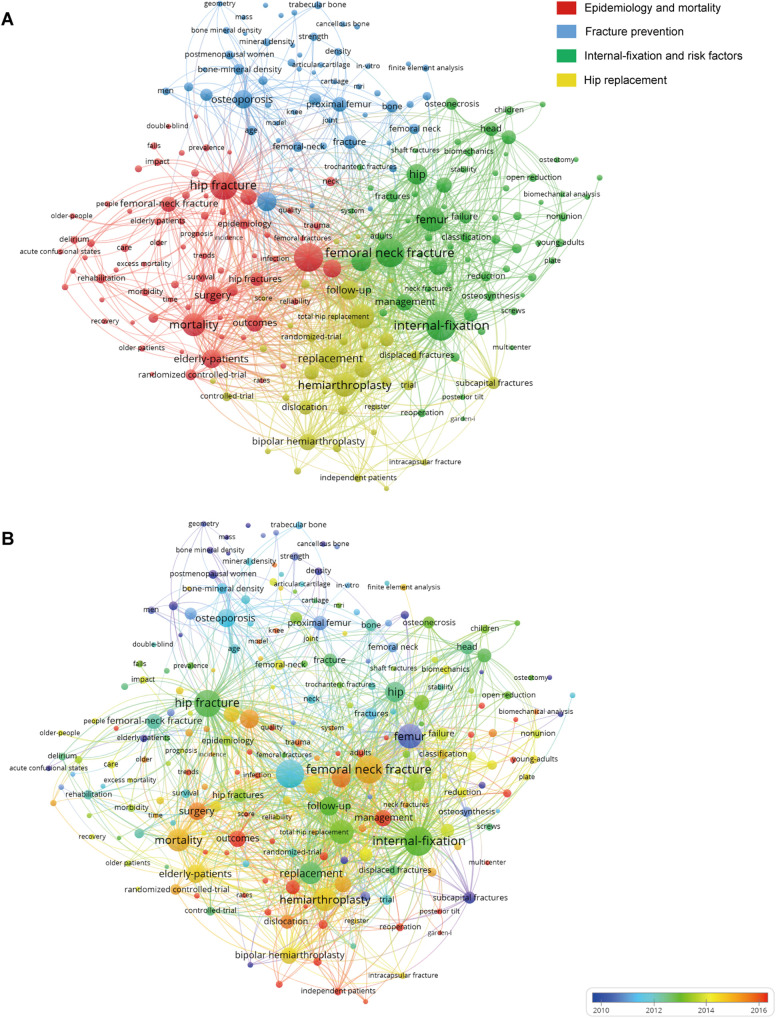
(**A**) Network visualization map of co-occurring keywords related to FNF research created by VOSviewer. (**B**) The overlay visualization map of the keyword co-occurrence analysis using VOSviewer.

A total of 7,863 keywords were extracted from 3,553 publications. As shown in Figure 6, the density visualization map was displayed with 230 keywords that occurred more than 20 times by using VOSviewer. Several hotspot clusters related to “femoral neck fracture,” “hip fracture,” “internal-fixation,” “replacement,” and “mortality” were observed. In terms of co-occurrence keyword clustering, all of them could be classified into four clusters in Figure 7A: Cluster 1 (“epidemiology and mortality,” red nodes); Cluster 2 (“fracture prevention,” blue nodes); Cluster 3 (“internal-fixation and risk factors,” green nodes); Cluster 4 (“hip replacement,” yellow nodes). In addition, we provided an overlay visualization map of co-occurrence keywords (Figure 7B). Different colors were applied for each keyword according to their average appearing year in articles.

**Figure 8:**
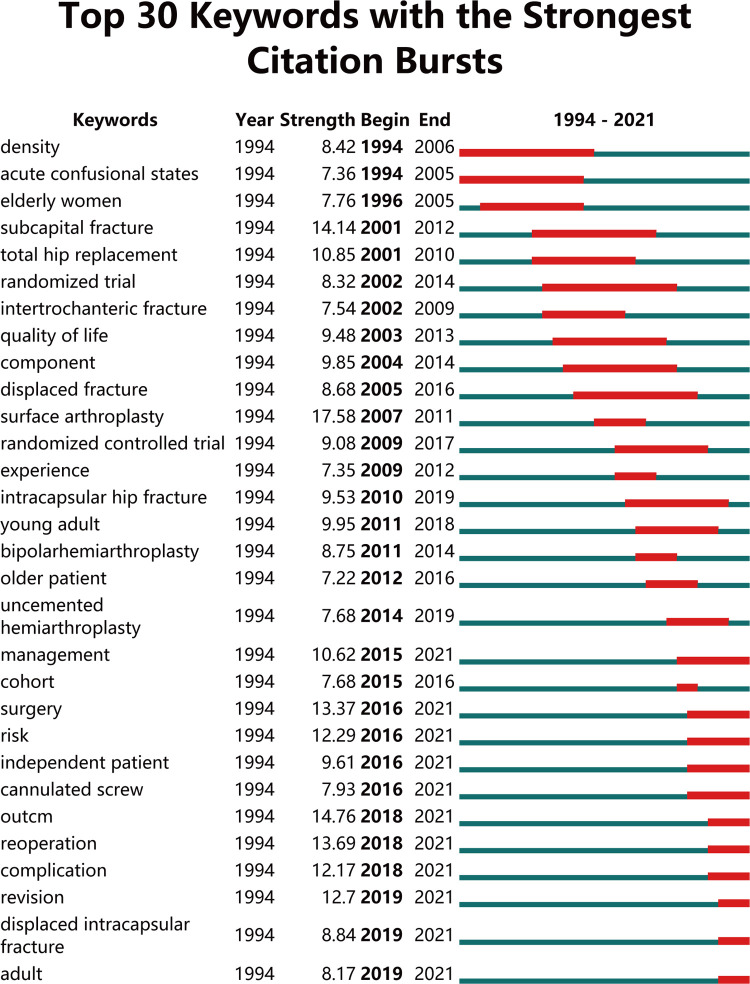
The top 30 keywords with the strongest citation bursts from 1994 to 2021 (generated by CiteSpace).

Moreover, burst keywords were regarded as another effective indicator of research hotspots, predicting the emerging tendencies to a certain extent. In this study, we applied the burst detection algorithm to extract keywords for FNF research. Figure 8 illustrated the top 30 keywords with the strongest citation bursts from 1994 to 2021. Among the whole list with the strongest citation bursts, “surface arthroplasty,” “outcome,” “subcapital fracture,” and “reoperation” were the top four keywords with the strongest burst strength (17.58, 14.76, 14.14, and 13.69, respectively). Notably, we also found that “revision,” “displaced intracapsular fracture,” and “adult” were the latest keywords that emerged in the last 3 years.

## Discussion

In this study, we presented a comprehensive overview of the knowledge framework, research hotspots, and theme trends in FNF research. Over the past 27 years, the number of publications of FNF showed a steady growth year by year. A total of 3,553 articles related to FNF were identified, and the number has increased approximately 7-fold since 1994. A steady growth in the article number indicates that FNF is not yet to be fully understood and is expected to be a research hotspot in the future.

### General Knowledge Framework of FNF

Among the top 10 countries, the USA has an absolute advantage, reflecting in the largest number of publication outputs and the highest value of H-index. In the early stages, the USA occupied the dominant position due to superior conditions of basic research and clinical trials. Nevertheless, the gap is gradually narrowed as the growing interest in Asian and European countries in this research field. The increasing trend in these countries may be explained by economic factors (25). The distribution of the top 10 related funding agencies also confirmed this situation. Of these, five funding institutions in the United States provided funding for publications of the FNF research. The USA is also a central collaborator, with extensive international cooperation with China, Canada, Japan, and the UK. In terms of ACI, ACI in the Netherlands exceeded other countries, ranking first, followed by Australia, Sweden, and the UK. The result might be related to the early appearance and high quality of the articles in these countries.

Sweden, the US, and Canada share eight out of the ten top research institutions. These results implied that first-class research institutions are critical for improving a country’s academic standards. Notably, despite a decent number of publications in Shanghai Jiao Tong University, the ACI was much lower than other institutions. Therefore, while pursuing the number of publications, attention should be paid to ensure the quality of research articles. Moreover, the collaboration visualization map indicated that the inter-institutional collaboration was relatively low. Although some Asian countries have contributed to the number of publications, there was no cooperation network between institutions in these regions. In addition, the McMaster University, Karolinska Institutet, Mayo Clinic, and University of Minnesota were the only four institutions with the BC values greater than 0.1, which means that other institutions do not yet have a strong influence on the field. Therefore, there is an urgent need to improve collaborations and knowledge communication in different institutions.

In terms of the journals, the *Injury International Journal of the Care of the Injured*, *Journal of Orthopaedic Trauma*, *Clinical Orthopaedics and Related Research*, *Journal of Arthroplasty*, and *Archives of Orthopaedic and Trauma Surgery* have published approximately one-fourth of all publications in FNF research. It can be speculated that future findings in this field will be published in the listed journals. Moreover, the *Journal of Bone and Joint Surgery-American Volume* has the largest impact factor and the highest value of ACI, indicating that this influential journal is more likely to publish high-quality researches in the future. These findings are consistent with other bibliometric studies of hip fractures (26). The dual-map overlay of FNF research shown that all the publications mainly targeted journals in two fields: (i) medicine, medical, and clinical; (ii) neurology, sports, and ophthalmology. The most-cited publications originated from the journals of (i) molecular, biology, and genetics; (ii) health, nursing, and medicine; (iii) sports, rehabilitation, and sport.

Cooperation and co-citation analysis could provide information for scholars to understand the influential authors and the existing partnerships in the FNF research field. Our results showed that Bhandari M, Schemitsch EH, Frihagen F, Parker MJ, and Rogmark C were the core authors in this field. For example, Bhandari M published the largest number of papers with the highest H-index in this area. His studies are broadly focused on clinical trials, meta-analyses, methodological aspects of surgery trials, and the translation of evidence into surgical practice. He has conducted many of the large and definitive surgical randomized trials in patients with FNF (27–29). Professor Frihagen F is one of the top experts in the field of FNF from the Ulleval University Hospital. The chief contribution of him was providing a great deal of clinical research data on hemiarthroplasty for FNF (30, 31). The major achievement of Professor Parker MJ was elaborating the incidence of fracture-healing complications after FNF (32, 33) and suggesting that regional anesthesia for hip fracture surgery is associated with a reduced early mortality and incidence of deep vein thrombosis in comparison with general anesthesia (34). While the main contribution of Professor Rogmark C was that he has done a great deal of clinical research and accumulated much scientific data on displaced FNF (35, 36). Furthermore, in the clustering analysis, “osteoporosis,” “total hip arthroplasty,” “subtrochanteric fracture,” “biomechanics,” “dxa,” and “prosthesis” contained the largest authors group, which indicated that these research topics obtained the most attention.

### Research Hotspots of FNF

Epidemiology and mortality: The incidence of FNF is increasing dramatically as the mean age of the population increases (37). In the United States, more than 250,000 hip fractures occur each year, with associated health care costs of $8.7 billion (38). In Sweden, fractures of the femoral neck constitute 53% of all hip fractures according to SAHFE (Standardized Audit of Hip Fractures in Europe) (39). Another study described the epidemiology of FNF in Italy (40). A total of 41,354 admissions for FNF were recorded, 75% of which were in females and the mean age of patients was 78 years. Regarding mortality, a systematic review of 70 trials found that mortality rates for FNF patients were similar over a 31-year period (∼20%), whereas another review reported that mortality rates could range from 14% to 58% within 1 year of fracture (41, 42). Kurtinaitis J et al. have followed 736 cases of FNF for 2 years, and found that the 1- and 2-year overall survival rates were 77.4% and 67.1%, respectively (43). Shah SN and colleagues have investigated the in-hospital mortality of 173,508 elderly patients with FNF from the Nationwide Inpatient Sample (NIS) database (44). He concluded that the in-hospital mortality after hemiarthroplasty for FNF was 3.1%. Postoperative complications, including pulmonary embolism, wound infection, and pneumonia, increased mortality risk by 4.59, 3.10, and 3.78 times, respectively.

Fracture prevention: The most-cited literature was published by Paul Lips in 2001 describing that Vitamin D3 supplementation causes a decrease of bone turnover and an increase of bone mineral density, which may decrease the incidence of hip fractures in nursing-home residents (45). Another high cited report suggesting that a combination of the measurement of BMD and bone resorption may be useful to assess the risk of hip fracture in elderly women (46). Osteoporosis remains the most important contributing factor to FNF, advances in the prevention and treatment of osteoporosis may finally decrease the incidence of these fractures (38).

Internal-fixation and risk factors: Currently, the available techniques for fracture fixation mainly included: cannulated screws, hip screw systems, proximal femur plates (PFP), cephallomedullary nails (CMN), and the femoral neck system (FNS) (47, 48). However, treatment of young FNF patients remains a big challenge because of the high rates of fracture complications. Avascular necrosis and nonunion were the most common complications that likely contributed to secondary surgery (49). Thus, recognizing the factors for predicting surgical effects is critical for the prevention of serious complications. Gumustas S et al. investigated factors that affect FNF in young adults in a retrospective clinical study (50). They found that the surgical timing and capsulotomy made no difference to the clinical results of FNF, and more serious fracture displacement was related to higher rates of complication. A multicenter study from Malaysia reported that the complications were associated with the mechanism of injury, capsulotomy, and type of fixation (51). In addition, various studies have investigated the metabolic and nutritional parameters factors related to fracture-healing (52–54).

Hip replacement: A meta-analysis reported that arthroplasty, including total hip arthroplasty and hemiarthroplasty, for the treatment of displaced FNF significantly reduces the risk of revision surgery compared to internal-fixation (55). A randomized control trial study noted that hemiarthroplasty and total hip arthroplasty likely resulted in similar clinical function, rates of revision, mortality, and dislocation at up to 5 years (56). Wang Z et al. (57) assembled a cohort of 70,242 patients with FNF and measured the incidences of dislocation and mortality after receiving a hip replacement. In contrast, he reported that patients treated with hemiarthroplasty after FNF had a significantly lower proportional hazard of reoperation than those treated with total hip arthroplasty. Overall, hemiarthroplasty and total hip arthroplasty have their own advantages in different aspects of outcomes including revision rate, mortality, quality of life, function, complications, cost-effectiveness, hospital stay, and surgical time.

### Changing Trends of FNF

As displayed in Figure 7B, all the nodes were noted with different colors according to the average appearing year. A trend of balanced development existed in the clusters of “fracture prevention” and “internal-fixation and risk factors,” over the past 27 years. In contrast, the clusters of “epidemiology and mortality” and “hip replacement study” have attracted increasing attention since 2014. Meanwhile, the other two clusters were also experiencing different degrees of development changes in FNF research hotspots. Burst keywords are often regarded as an indicator of research hotspots, predicting the emerging trend in the special field. Moreover, we have analyzed the top 30 keywords with the strongest citation bursts from 1994 to 2021. It can be seen from the changing trend of burst keywords in different periods, the treatment of FNF in the elderly has always been a hotspot. Notably, from 2011 to 2021, the number of studies related to treatment of young patients is increasing. Topics such as complications and surgical challenges of FNF in young adults are drawing orthopedics attention. Over the recent 4 years, from 2018 to 2021, the most frequently encountered keywords were “outcome,” “reoperation,” “complication,” “revision,” “displaced intracapsular,” “fracture,” and “adult,” and the bursts are still ongoing. This result indicated that these research directions have a considerable potential to continue to be the research hotspots and focus in the near future.

## Strengths and Limitations

Our study used bibliometric and visual analyses to assess the knowledge framework, research hotspots, and theme trends in the field of FNF research. However, several limitations need to be acknowledged in our study. Firstly, we only derived the publications from a single WoSCC database and neglected the other large databases, which could miss a few relevant literature inevitably. However, as mentioned in previous studies, WoSCC was the most popular used database for bibliometric analysis (24, 26). Furthermore, the data from WoSCC were adequate to reflect the current state of FNF research. Secondly, an unavoidable limitation of the bibliometric analysis was the potential for incomplete searches of studies due to the restriction of the search terms. This may partially affect the precision of the results but is unlikely to change the final conclusions. Thirdly, only English-language publications were included in the final analysis, which may cause language bias.

## Conclusion

This study presented a comprehensive overview of the knowledge framework and research hotspots in FNF research from 1994 to 2021 and predicted future theme trends in this field. It can be predicted that the number of publications on FNF research will increase, and the United States maintain a leading position in this field. The global distribution of FNF research is uneven, and collaboration and knowledge communication between institutions and authors need to be improved. The management of FNF in young patients is drawing more attention from orthopedic surgeons, it is expected that these research topics may continue to be the research hotspots and focus in the near future.

## Data Availability

The original contributions presented in the study are included in the article/supplementary material, further inquiries can be directed to the corresponding author/s.
